# Dopamine in the Pathophysiology of Preeclampsia and Gestational Hypertension: Monoamine Oxidase (MAO) and Catechol-O-methyl Transferase (COMT) as Possible Mechanisms

**DOI:** 10.1155/2019/3546294

**Published:** 2019-11-28

**Authors:** Wendy N. Phoswa

**Affiliations:** Department of Life and Consumer Sciences, University of South Africa (UNISA), Science Campus, Private Bag X6, Florida, Roodepoort 1710, South Africa

## Abstract

**Purpose of the Review:**

Hypertension in pregnancy is the global health burden. Amongst the hypertensive disorders of pregnancy, preeclampsia and gestational hypertension are the world's leading disorders that lead to both maternal and fetal morbidity and mortality.

**Recent Findings:**

Dopamine inactive metabolites, namely, monoamine oxidase (MAO) and catechol-O-methyl transferase (COMT), have been reported to be associated with hypertensive disorders of pregnancy such preeclampsia and gestational hypertension.

**Summary:**

This review discusses the involvement of MAO and COMT in the pathophysiology of both conditions in order to have a better understanding on the pathogenesis of both conditions, suggesting promising therapeutic interventions and subsequently reducing maternal and fetal morbidity and mortality.

## 1. Introduction

Apart from preeclampsia (PE) which accounts 70% of all hypertensive cases of pregnancy, gestational hypertension (GH) is also one of the life-threatening illnesses associated with hypertensive disorders of pregnancy (HDP). Gestational hypertension, also known as pregnancy-induced hypertension (PIH) without proteinuria, makes up to 10% of HDP with significant variations in certain parts of the world depending on diagnostic criteria [1].

Preeclampsia is defined as elevated blood pressure (SBP ≥ 140 mmHg or DBP ≥ 90 mmHg) after 20 weeks of gestation in a previously normotensive woman [2] without proteinuria. Both PE and GH have similar risk factors (e.g., high BMI, type 1 diabetes, and gestational diabetes) which contribute to the pathogenesis of the disease.

There is still ongoing debate as to whether PE which is associated with elevated protein excretion is a different condition to nonproteinuric hypertension (gestational hypertension) or whether it is indeed a different part of a spectrum of the same disease [2, 3]. Although the pathophysiology of GH and PE has been reported to emanate from increased oxidative stress which results from reduced placental perfusion followed by exaggerated maternal inflammatory response and endothelial dysfunction [4–6], the exact pathophysiology that leads to the clinical features of both conditions still remains undefined.

Over the last decade, substantial progress has been made in understanding the pathophysiology of both conditions. Recent reports point towards the dopamine bioavailability. It has been reported that altered levels of dopamine production may lead to a number of pathologies including oxidative stress, edema, and either genetic or essential hypertension [7].

## 2. Dopamine-Induced Oxidative Stress

Dopamine has been reported as one of the major sources of oxidative stress. This oxidation occurs through the activity of an enzyme known as prostaglandin H synthase [8] or with mitochondrial proteins [9]. Additionally, dopamine induces oxidative stress via monoamine oxidase (MAO) activity [10, 11].

Dopamine-induced oxidative stress has been implicated to be involved in aging and neurodegeneration disorders such as schizophrenia and Parkinson disease [12–15]. A study conducted by Grima et al. showed that dopamine decreases glutathione by 40% [12]. Glutathione is an antioxidant that plays a crucial role in protecting the cells from damage by reactive oxygen species generated by dopamine metabolism [12].

## 3. Dopamine Metabolism

Dopamine is broken down into inactive metabolites by a set of enzymes—monoamine oxidase (MAO), catechol-O-methyl transferase (COMT), and aldehyde dehydrogenase (ALDH). Both MAO and COMT have been found to play a role in normal placental development, and the absence or excessive production of these enzymes has been associated with hypertensive disorders of pregnancy [16–18].

Monoamine oxidase (MAO) is an enzyme involved in the oxidative deamination of amine neurotransmitters, including noradrenaline, serotonin, and dopamine, and exists as two isoenzymes, MAO-A and MAO-B. These enzymes differ in substrate specificity [19, 20] and tissue expression. MAO-A is predominant in the placenta compared to MAO-B which is present at low levels [21]. MAO-B enzyme is also present in platelet and lymphocytes [22].

## 4. The Role of Monoamine Oxidase (MAO) in the Pathophysiology of Hypertensive Disorders of Pregnancy

In the placenta, MAO has been reported to play an essential role in protecting the fetus since MAO inhibition has been found to lead to fetal growth restriction and pregnancy loss [23–25]. Interestingly, it has also been suggested that MAO is involved in the regulation of fetomaternal blood flow [26]. Although studies have indicated the importance of MAO in normal pregnancy, there is currently no data reported in relation to the role of MAO in hypertensive disorders of pregnancy. Therefore, more studies are needed in order to understand the role of this enzyme in the pathophysiology of hypertensive disorders of pregnancy. Current studies have however reported on the role of this enzyme to be associated with endothelial dysfunction [27, 28].

### 4.1. Monoamine Oxidase-Induced Endothelial Dysfunction

Endothelial dysfunction is one of the factors that lead to the pathogenesis of both PE and GH [29, 30]. MAO has been reported as a mediator for endothelial dysfunction [31]. Several studies have reported on the endothelial dysfunction induced by MAO [32, 33]. A study conducted by Sturza et al. reported that MAO-A and MAO-B contribute to the development of endothelial dysfunction through the activation of reactive oxygen species in the mouse aorta [27]. Similarly, Sun et al. reported that increased MAO-A expression in endothelial cells and cardiomyocytes contributes to vascular dysfunction and left heart failure [28].

Additionally, Sturza et al. showed that MAO induced endothelial dysfunction by increasing reactive oxygen species (ROS) in diabetic rats [34]. Similar findings were previously observed by Kluge et al. [33]. Interestingly, inhibition of MAO has been shown to successfully improve endothelial function and various studies have supported this finding. Sturza et al. showed that MAO inhibition can potentiate restoring endothelium-dependent relaxation in an experimental model of hypertension in a rat [34]. Furthermore, they also demonstrated that MAO-A inhibition improves endothelial dysfunction in Zucker diabetic fatty (ZDF) rat, a genetic model of type 2 diabetes [32]. Similarly, Lighezan et al. reported that MAO inhibitors restore endothelial function in conditions associated with increased oxidative stress [35].

### 4.2. Monoamine Oxidase-Induced Oxidative Stress

Monoamine oxidase (MAO) is also known as the primary source of oxidative stress [36]. MAO has been reported to play a role in the pathophysiology of hypertensive disorders of pregnancy [37]. This occurs as a result of increased oxidative stress induced by MAO. However, inhibition of MAO activity has been reported to reduce the vascular formation of reactive oxygen species, (H_2_O_2_), and partially leads to improved endothelium-dependent relaxation in vessels preexposed to angiotensin II and lipopolysaccharide [27].

Oxidative stress is the major factor involved in the pathophysiology of both PE and GH. Several studies have reported that there is increased oxidative stress in both conditions [38–42].

MAO-induced oxidative stress occurs when a FAD cofactor catalyses the oxidative deamination of several monoamines (e.g., serotonin, norepinephrine, and dopamine) and exogenous amines such as tyramine, generating H_2_O_2_, aldehydes, and ammonia as by-products [36]. Since MAO is divided into MAO-A and MAO-B, amongst neurotransmitters oxidized by MAO, serotonin oxidation is only catalysed by MAO-A, and norepinephrine, dopamine, epinephrine, and tyramine oxidization is catalysed by both MAO-A and MAO-B. Additionally, oxidation of phenylethylamine is catalysed by MAO-B [36, 43].

Several studies have reported on the role of these neurotransmitters in the pathophysiology of cardiovascular diseases (e.g., hypertension) [44–54].

#### 4.2.1. Serotonin

High serotonin levels have been reported to play a role in the pathogenesis of cardiovascular diseases and hypertension [44–54]. A study conducted by Aflyatumova et al. looking at endothelin-1, nitric oxide, and serotonin in male adolescents showed that both endothelin-1 and serotonin serum concentration levels were increased in prehypertensive and hypertensive individuals compared to controls. They also observed increased levels of NO in prehypertensive individuals compared to controls and hypertensive individuals [54]. Serotonin has also been implicated to play a role in hypertensive disorders of pregnancy [55–57]. Recently, it has been reported that women exposed to serotonin therapy had risk of preeclampsia and gestational hypertension [58]. Poulson et al. suggested that serotonin plays a role in the pathophysiology of preeclampsia [55–57]. These findings were verified by Senior et al., who observed elevated levels of serotonin in the placentas of preeclamptic patients compared to controls [59]. Other studies have reported that there is an increase in the urinary excretion of serotonin metabolites in preeclampsia [60, 61]. In contrast, Lupattelli et al. showed that pregnant women exposed to serotonin had no increased risk of PE [62]. Pathophysiological mechanism underlying the association between serotonin and preeclampsia is unclear. However, we speculate that the underlying mechanism involves increased endothelin-1 (ET-1) and reduced nitric oxide (NO) levels.

Both ET-1 and NO have been reported to play a role in hypertensive disorders of pregnancy [63–68]. Endothelin-1 and NO are located in the endothelium and they play different roles. Endothelin-1 acts as a vasoconstrictor and NO inhibits the expression of adhesion molecules and platelet aggregation and acts as a vasodilator [69]. Interestingly, serotonin also acts as a vasoconstrictor which makes it possible that inducing AT-1 might lead to decreased NO and increased serotonin levels which leads to increased blood pressure.

Currently, there is very limited data associating serotonin levels and the risk of gestational hypertension. Therefore, more studies are needed to confirm whether serotonin exposure during pregnancy leads to adverse effects or not.

#### 4.2.2. Adrenaline

Catecholamines such as noradrenaline and adrenaline have also been implicated in the pathophysiology of hypertension and hypertensive disorders in pregnancy [70–72]. A study conducted by øian et al. showed that arterial adrenaline was associated with mean arterial blood pressure in preeclamptic patients [73]. However, their findings were in contrast with those of a previous study by Pedersen et al., who observed no significant difference between adrenaline and noradrenaline levels of preeclamptic compared to normotensive women [74]. There is also very few data reporting on the association between catecholamines and hypertensive disorders of pregnancy. More studies are needed in order to see how they are regulated in the presence of PE or GH.

#### 4.2.3. Norepinephrine

**Figure 1:**
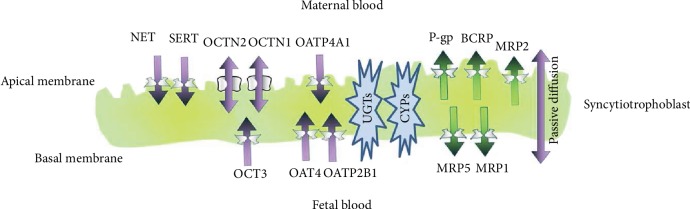
A schematic diagram showing norepinephrine transporter (NET) in the syncytiotrophoblast layer of the placenta. Norepinephrine from the maternal circulation enters the placenta and is transported to the fetal blood by NET [80].

Norepinephrine is another type of catecholamine released during pregnancy. In normal pregnancy, the placenta expresses norepinephrine transporters (NETs) that are responsible for maintaining normal fetal circulation and fetomaternal exchange (Figure 1) [75]. It has been reported that the NETs are expressed at minimal amounts in preeclamptic pregnancies [76]. A study conducted by Na et al. reported a reduced NET mRNA expression in preeclamptic placentas compared with normal placentas. More interestingly, they also observed that maternal plasma NE concentration was increased in preeclamptic women compared to normal pregnant women [77]. Similar findings were observed by Lampinen et al., who also noted an increase in plasma levels of norepinephrine in women with a previous history of PE [78]. Since catecholamines also accumulate in platelets, association between increased platelet NE and the risk of preeclampsia was reported by O'Shaughnessy et al. [79].  Table 1 indicates a comprehensive list of studies in this review examining the role of neurotransmitters oxidized by monoamine oxidases (MAO) in hypertensive disorders of pregnancy.

## 5. The Role of Catechol-O-methyl Transferase (COMT) in the Pathophysiology of Hypertensive Disorders of Pregnancy

Catechol-O-methyl transferase (COMT) is a key enzyme involved in catecholamine and estrogen degradation [81], and it was found to be active in both the placenta and the decidua [82]. Catechol-O-methyl transferase (COMT) has been reported to be involved in trophoblast invasion [83]. Reduced COMT bioavailability has been reported to be associated with hypertensive disorders of pregnancy [84, 85]. A study conducted by Kanasaki et al. reported that pregnant mice deficient in COMT developed multiple functional and structural features of preeclampsia-like phenotype due to the absence of 2-ME which is a metabolite of 17*β*-estradiol generated by COMT [86]. Similarly, Lai et al. observed decreased expression of COMT in the placentas from term preeclamptic patient [18]. In contrast, a study conducted by Palmer et al. reported that there was no significant difference in placental COMT expression in preeclamptic women compared to normotensive women. Their findings suggested that preeclampsia may not be associated with a decrease in placental COMT expression [87]. More studies are needed to confirm how COMT is regulated in the presence of PE in order to have a better understanding on the pathophysiology of the disease.

**Figure 2:**
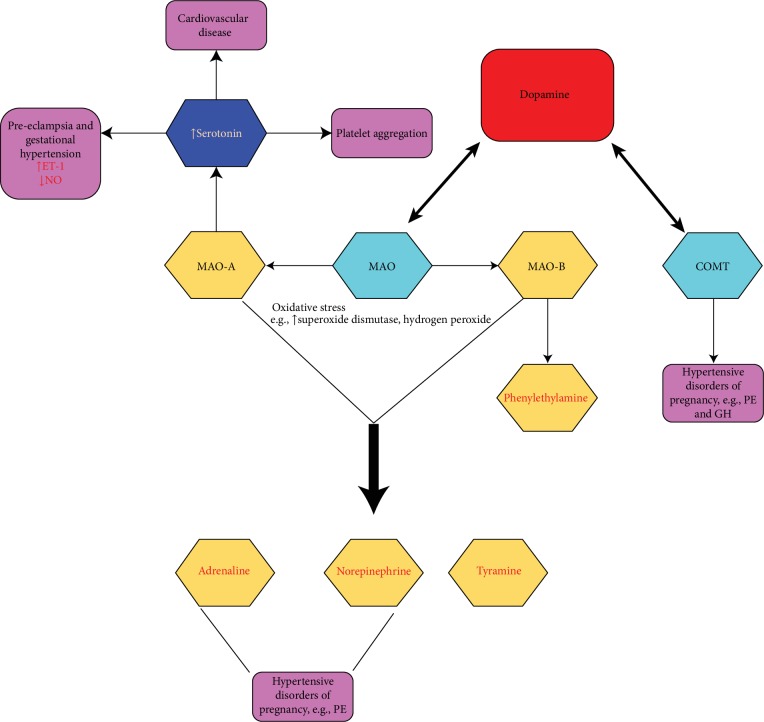
Schematic showing the role of dopamine-induced oxidative stress in the pathophysiology of hypertensive disorders of pregnancy such as PE and GH.

A number of studies highlighted the role of COMT in gestational hypertension [88, 89]. A recent study done by Hernandez et al. reported that the inhibition of COMT was associated with reduced NO bioavailability which resulted in endothelial dysfunction in GH [90]. However, more studies are needed to investigate the COMT mechanisms involved in the pathophysiology of both PE and GH.  Table 2 shows a summary of studies that have been done to date examining the role of catechol-O-methyl transferase (COMT) in the pathophysiology of hypertensive disorders of pregnancy, and Figure 2 summarizes the role of dopamine in the pathophysiology of hypertensive disorders of pregnancy such as PE and GH.

## 6. Conclusion

MAO and COMT are dysregulated in the presence of both PE and GH. More research is needed to investigate how these enzymes are regulated in the presence of each disorder in order to help develop effective antihypertensive drugs that can inhibit or stabilize the levels of these enzymes in pregnancy. This will help in improving prenatal diagnostic procedures and reducing maternal and fetal death rates.

## Figures and Tables

**Table 1:** A comprehensive list of studies in this review examining the role of neurotransmitters oxidized by monoamine oxidases (MAO) in hypertensive disorders of pregnancy.

Author	Neurotransmitters	Main findings
[60, 61]	Serotonin	Increase in the urinary excretion of serotonin metabolites in preeclampsia
[59]		Elevated levels of serotonin in the placentas of preeclamptic patients compared to controls
[62]		Pregnant women exposed to serotonin had no increased risk of PE.
[73]	Adrenaline	Associated with mean arterial blood pressure in preeclamptic patients
[76]	Norepinephrine	NETs are expressed at minimal amounts in preeclamptic pregnancies.
[77]		NE concentration is increased in preeclamptic women compared to normal pregnant women.
[78]		Increase in plasma levels of norepinephrine in women with previous history of PE

**Table 2:** A comprehensive list of studies in this review examining the role of catechol-O-methyl transferase (COMT) in the pathophysiology of hypertensive disorders of pregnancy.

Author	Country	Design	Cohort size	Main findings
[32]	United States	Cohort	270 normal women 284 women with hypertension	COMT activity is low in patients with hypertension.
[86]	United States	Review	Preeclamptic women	Deficiency in catechol-O-methyl transferase and 2-methoxyoestradiol is associated with preeclampsia.
[87]	Australia	Cohort	14 healthy term pregnant women 8 preterm normotensive pregnancy 22 severe preeclamptic women	Severe preeclampsia may not be associated with a decrease in placental COMT expression.
[18]	China	Cohort	15 normal pregnant women 15 term pregnant patients with preeclampsia	COMT may play a role in the pathogenesis of term preeclampsia.
[90]	Spain	Cohort	Pregnant Sprague–Dawley rats	COMT is associated with reduced NO bioavailability which results to endothelial dysfunction in GH.
